# Facing a Clever Predator Demands Clever Responses - Red-Backed Shrikes (*Lanius collurio*) vs. Eurasian Magpies (*Pica pica*)

**DOI:** 10.1371/journal.pone.0159432

**Published:** 2016-07-25

**Authors:** Michaela Syrová, Michal Němec, Petr Veselý, Eva Landová, Roman Fuchs

**Affiliations:** 1 Department of Zoology, Faculty of Science, University of South Bohemia, Branišovská 1760, České Budějovice, 37005, Czech Republic; 2 Department of Ethology, Institute of Animal Science in Prague, Přátelství 815, Prague – Uhříněves, 10400, Czech Republic; 3 Department of Zoology, Faculty of Science, Charles University in Prague, Viničná 1594/7, Praha – Nové Město, 12800, Czech Republic; Hungarian Academy of Sciences, HUNGARY

## Abstract

Red-backed shrikes (*Lanius collurio*) behave quite differently towards two common nest predators. While the European jay (*Garrulus glandarius*) is commonly attacked, in the presence of the Eurasian magpie (*Pica pica*), shrikes stay fully passive. We tested the hypotheses that this passive response to the magpie is an alternative defense strategy. Nesting shrikes were exposed to the commonly attacked European kestrel (*Falco tinnunculus*) in a situation in which i) a harmless domestic pigeon, ii) a commonly attacked European jay, and iii) a non-attacked black-billed magpie are (separately) presented nearby. The kestrel dummy presented together with the magpie dummy was attacked with a significantly lower intensity than when it was presented with the other intruders (pigeon, jay) or alone. This means that the presence of the magpie inhibited the shrike’s defense response towards the other intruder. These results support our previous hypotheses that shrikes use an alternative defense strategy in the magpie’s presence. We hypothesize that the magpie is able to associate the active defense of the shrikes with the close proximity of a nest and that shrikes try not to draw the magpie’s attention to the nest. The reason why this strategy is not used against the jay remains unanswered as jays as well as magpies show very similar cognitive and foraging skills enabling them to individuate the nest presence according to active parental defense.

## Introduction

Predator recognition and categorization is an essential cognitive ability enabling the optimization of antipredator behavior [1][2]. Prey species may ignore the presence of a less dangerous predator [3], while it must choose the appropriate antipredator behavior towards the specialized predators of adults or nests [4][5][6][7].

The red-backed shrike (*Lanius collurio*) shows a vigorously active nest defense behavior towards various predators and nest parasites [8][9] including humans [10]. In our previous study [8] we tested shrikes’ responses to two corvid nest predators (Eurasian magpie *Pica pica* and European jay *Garrulus glandarius*). While the jay was commonly attacked, in the presence of a magpie shrikes stayed fully passive; despite the threat represented by both corvid species being equal at first sight. They are both common nest predators of similar size [11]. Although, there is evidence of differences in the intensity of defense responses towards various birds of prey (e.g. [8][12]), owls (e.g. [13]), or corvids (e.g. [14]), such a qualitative difference in the antipredator response towards two members of the same predator guild has never been shown.

In our previous study, we suggested that a likely explanation for the passive response of the shrikes to the magpie is that it is a strategy designed not to draw attention to their nest rather than an absence of interest [8]. There is little evidence for such behavior (e.g. [15][16][17]) because it is not easy to show that the absence of response is an alternative strategy. A more often described alternative antipredator strategy is some form of *distraction display* ([18][19][20][21][22][23][24][25]; summarized in [2]).

In the present study, we tried to test the hypothesis that magpies are not attacked by shrikes, because they use an alternative antipredatory strategy against it. We exposed shrikes to a magpie together with another commonly attacked predator. This situation induced a multiple predator conflict (indexed as MPC hereafter). An MPC constitutes a situation in which a specific defense response towards one predator may put the prey at greater risk of being threatened by the other predator [26][27][28]. In this case, the best solution of the MPC is to choose the response optimal for the more dangerous predator (reviewed [29]). If the goal of the shrikes’s passive behavior in the presence of a magpie is not to draw attention to the nest, an active response to another predator in the presence of the magpie would be counterproductive. Assuming the shrikes regard the magpie as a greater danger than any other predator, the best solution of MPC would be passive behavior.

We exposed the shrikes to two pairs of intruders: a European kestrel (*Falco tinnunculus*, a commonly attacked, less dangerous predator of fledglings and adults) with a magpie (a non-attacked nest predator) and with a jay (a commonly attacked nest predator). We tested the hypothesis presuming that the shrikes’ passive behavior is an alternative defense strategy and that shrikes consider the magpie as a greater danger than predators which are usually attacked. In this case the shrikes would attack the kestrel less in the presence of the magpie than in the presence of jay or a harmless bird species (control).

## Methods

### Study area

The study took place in the Doupov mountains, near the town of Karlovy Vary (Western Bohemia; 50°10´N, 13°9´E), the Czech Republic. Experiments were conducted during the breeding season (from early June to late July) in the years 2011 and 2012 and between 10:00 and 18:00.

### Study species

The studied species, the red-backed shrike, is a medium sized passerine bird. However it uses active mobbing, including physical attacks, as an important part of its antipredator behavior [8][10].

We chose the dummy of a small bird of prey, adult female European kestrel, as the intruder immediately endangering the nest. The kestrel was the most attacked predator by the red-backed shrikes in our previous study [8].

The dummies of a magpie, jay, and domestic pigeon were chosen as the bystander intruders, which represent only a potential danger. The magpie and the jay are also similarly sized [11] and known as the common predators of passerine nests [30][31][32]. The domestic pigeon is a harmless jay- or magpie-sized bird. In our previous study [8], the red-backed shrikes attacked the jay intensively, while attacks against the magpie and the domestic pigeon occurred only exceptionally. Shrikes are familiar with all of these intruders ([33]; personal observation).

### Experimental design

**Fig 1:**
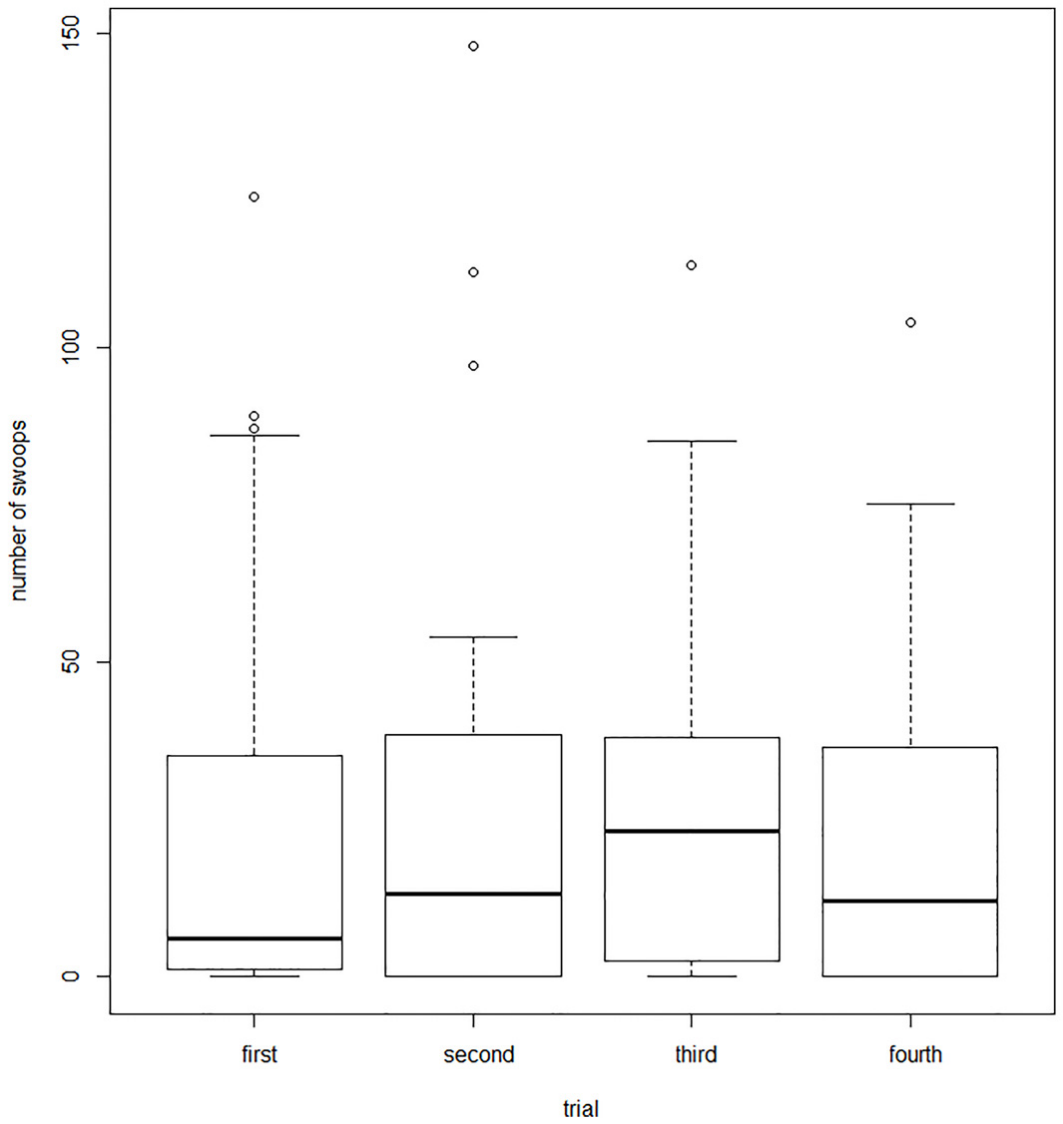
Number of swoops performed by shrikes against the kestrel dummies in particular trials. Line in the middle refers to median, box shows 25–75% of data, whiskers show 10–90% of data and dots are outliers.

Every pair of shrikes was successively tested in four trials: ‘the kestrel with the magpie’, ‘the kestrel with the jay’, ‘the kestrel with the domestic pigeon’, and ‘the kestrel alone’ (without the second intruder). The sequence of these trials was random. We did not show any effects of the order of presentation on shrike responses (Linear mixed effect model—indexed as LMM hereafter, F_242,3_ = 0.17, p = 0.99; Fig 1).

All dummies were placed in an upright position with their wings folded. The kestrel was placed 1m away from the nest on a 1.5 m high pole, facing the nest. The second intruder (magpie, jay, or domestic pigeon) was installed 10 m from the nest, also on a 1.5 m high pole facing the nest. We chose this distance on the basis of our previous research [34] showing that shrikes only seldom attack the jay 10m away from nest. We used four different dummy specimens of each species to avoid a bias caused by the dummy identity. All dummies were stuffed by the same taxidermist in the same position. The dummy identity had no effect on shrikes’ responses (kestrel: LMM, F_242,3_ = 1.27, p = 0.86; magpie: LMM, F_242,3_ = 0.97, p = 0.92; jay: LMM, F_242,3_ = 2.34, p = 0.71; pigeon: LMM, F_242,3_ = 0.11, p = 0.99). The defense behavior was taped on DV Camera (Panasonic HC-V510).

Each trial (presentation of dummies) lasted 20 minutes. The time interval between the trials was one hour allowing shrikes to calm down and supply food to their brood. During the years 2011 and 2012 we examined 20 nests with nestlings at an age of between 5 to 15 days. Female and male behaviors were analyzed separately. We recorded the occurrence of any swoops both with and without physical contact.

### Statistical analyses

We created linear mixed effect models (LMM) with the random slope model arrangement (random factor ‘individual ID’ nested in the random factor ‘pair ID’) to assess the effect of predictor variables (command lmer in R package lme4 [35]). The response variable was the *number of swoops* performed by each individual tested shrike against the kestrel dummy during one trial. In order to meet the demands of normal distribution these data were transformed by logarithmic transformation [log (no of swoops + 1)].

The main categorical predictor variable, the *type of bystander*, had the following four values: jay, magpie, pigeon, none. Other categorical predictors in the model were the *sex of the shrike* (values ‘male’ and ‘female’) and the *order of the trial* within the sequence (values ‘first’, ‘second’, ‘third’, ‘fourth’). There was also one continuous predictor variable: the *age of the nestlings*.

The effects of the predictor variables were evaluated using a likelihood ratio test based on Gaussian distribution and partial F-test. The Tukey HSD post-hoc tests were used to evaluate the differences among the levels of categorical predictors.

To rank the models, AICc values were computed, and from these the difference in AICc (ΔAICc) was calculated by subtracting the lowest AICc from all others. From this, as measures of strength of evidence for each model, the relative likelihood (exp (0.5/ΔAICc)) and the probability or Akaike weight (relative likelihood/sum of all relative likelihoods*10) were computed [36]. The models are shown in Table 1. The results of the model with the highest Akaike weight are presented in Results (marked with bold font in Table 1). All statistical analyses were computed in R 3.2.1 (R Development Core Team 2015).

**Table 1:** Model selection for the response variable from linear mixed effect models.

Response variable	Model	AICc	ΔAICc	Relative likelihood	Akaike weight
Log (no. swoops+1)	Intercept	385.74	32.7	1.02	0.58
	Bystander	353.04	0	1	0.57
	Age	386.63	33.59	1.01	0.58
	Sex	387.34	34.30	1.01	0.58
	**Bystander+age**	**354.13**	**1.09**	**1.58**	**0.90**
	Bystander+sex	354.63	1.59	1.37	0.78
	Age+sex	388.22	35.18	1.01	0.58
	Bystander+age+sex	355.7	2.66	1.21	0.69

Bold type indicates the best models, which were determined based on relative AICc values (ΔAICc) and computed relative likelihood and Akaike weights. Intruder—the type of the intruder, order—the trial order within the sequence, age—the age of the nestlings, sex—the sex of the parent shrike.

### Ethical note

This study was conducted in accordance with the valid laws and regulations of the Czech Republic; in compliance with the Ethic Committee of the Faculty of Science, University of South Bohemia, which approved this study. Behavioral experiments on the wild birds were enabled by accreditation no. 13842/2011-30 and a license permitting experimentation with animals no. CZ01629 offered by the Ministry of the Agriculture of the Czech Republic. We have observed that our activities influenced neither the life of the tested birds nor the fate of their nests. Moreover the density of nesting shrikes in the tested populations has been stable for the last 5 years.

## Results

**Fig 2:**
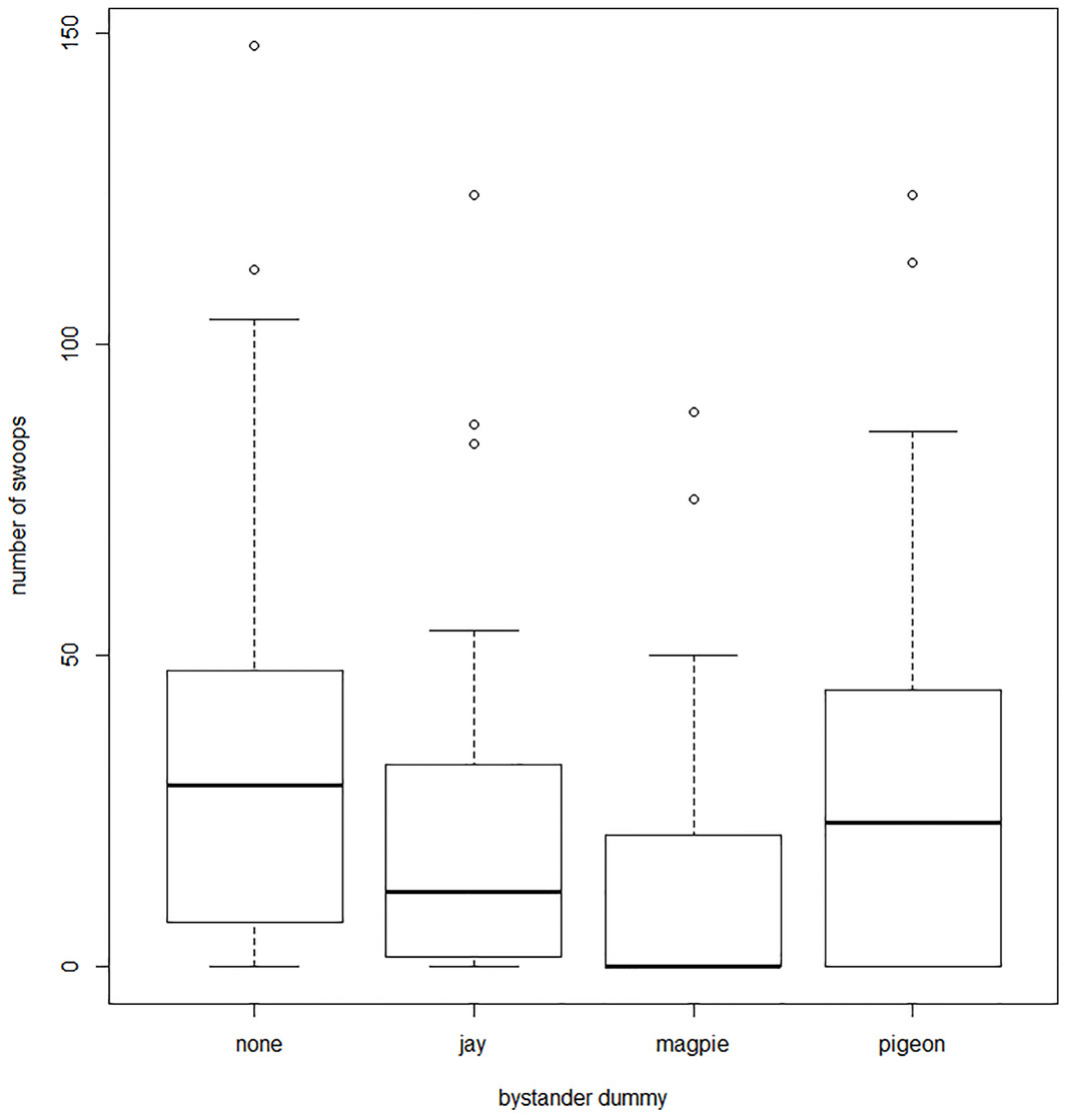
Number of swoops performed by shrikes against the kestrel dummies presented together with three bystander dummies (jay, magpie, pigeon) or alone during the 20-minutes lasting trial. Line in the middle refers to median, box shows 25–75% of data, whiskers show 10–90% of data and dots are outliers.

Only the *type of bystander* affected the number of swoops the shrikes performed against the kestrel (Fig 2, Table 2). Post hoc Tukey HSD tests showed that the kestrel presented with the magpie bystander was attacked less than kestrel presented together with the jay (z = -3.21, p<0.01), pigeon (z = -3,82, p<0.01) or alone (z = -6,21, p<<0.01). The *number of swoops* towards the kestrel in other trials did not differ (Tukey HSD test; kestrel with jay x kestrel with pigeon: z = -0.29, p = 0.98; kestrel with jay x kestrel alone: z = -2.53, p = 0.08; kestrel with pigeon x kestrel alone: z = -2.10, p = 0.10).

**Table 2:** Factors influencing intensity of mobbing (number of swoops) performed by shrikes against the kestrel (LMM).

	numDF	denDF	F-value	p-value
Intruder type	3	242	31.27	<0.01
Age of nestling	1	242	0.08	0.91

## Discussion

In concordance with our hypothesis, the kestrel dummy presented together with the magpie dummy was attacked with a significantly lower intensity than when it was presented with the other intruders (pigeon, jay) or alone. This means that the presence of the magpie inhibited the shrikes’ defense response towards the other intruder.

Our new results further show that 1) shrikes are able to solve the MPC arisen from the co-occurrence of a predator, towards which the active nest defense is effective; and a predator, towards which the active nest defense is not effective; 2) shrikes are able to solve this MPC although the predator towards which the active nest defense is effective represents a greater threat than the predator towards which the active nest defense is not effective.

Our results support the hypothesis that the passive behavior of shrikes in the presence of magpies is not an expression of indifference but an alternative defense strategy, and that shrikes perceive the magpie as being more dangerous than the kestrel. The results also concur with (but do not corroborate) our previous hypothesis [8] presuming that shrikes choose an alternative defense strategy against a magpie because active defense may draw attention to the nest. There is evidence for the suppression of active nest defense as an alternative antipredator strategy in birds ([37][38]), though the number of such studies is in striking contradiction to the fact that there is broad evidence in the literature that mobbing can attract a predator`s attention [39][40][41][42][43][44][45][46].

Unfortunately, based on our results, we cannot explain why shrikes consider active defense risky against a magpie and not against a jay. It has been proven in other bird species that they commonly drive jays away from the nest ([38][47][48], but see [49]), while in presence of a magpie parents avoid any interaction with it [50][51]. Nevertheless, in these studies, there are no comparisons with responses to other predators. There are a few alternative explanations for the different responses of shrikes towards jays and magpies which can be meditated.

Firstly, a magpie may devote greater effort to searching for nests. When compared to the diet of the jay, the diet of magpies is biased towards vertebrate prey including adult birds, small mammals, reptiles, and carrion [52][53]. Moreover, it has been documented that magpie predation can affect the nest success and density of songbirds [31][54][55][56][57], including shrikes [58]. Nevertheless, there is no evidence that magpies, rather than jays, are able to individuate the presence of the nest according to other signals e.g. the excitement of parents. It has been shown that predators have the ability to be attracted to the nest by parents’ alarm calls [44][45][46], but in such cases the predators are assumed to have developed spatial cognition and sometimes, to some extent, memory. Both these cognitive skills are quite well developed in magpies and jays. In general Corvids are more successful in mental and cognitive tests than other bird groups [59][60]. In laboratory tests the performance of jays and magpies in terms of long-term spatial memory (magpies [61], jays [62]) or object permanence (magpies [63], jays [64][65]) was almost equal.

Another parameter which eases the searching of predators for nests is the social system of magpies and jays [59][66][67]. Both of them live in family groups (magpie [30][68][69], jay [70]) and are able to obtain information in a social context [70][71][72][73][74][75].

Altogether, the difference between magpies and jays in terms of their ability to individuate the presence of a nest based on parental excitement is very small, at least from the human point of view.

Another potential explanation may reside in the different experience of shrikes with jays and magpies, probably in terms of evolutionary history rather than individually. Jays and magpies are ecologically very similar, both congregate in open landscape with fragmented forests, although jays are more specialized to forests and magpies to the open landscape [76]. Shrikes may thus have more evolutionary experience with the magpie, as they both are probably primarily birds of open habitats with scattered shrubs and trees.

This brings us to the question of how shrikes have obtained the ability to suppress active nest defense behavior. We may hypothesize several scenarios leading to the acquisition of such a skill: 1) The shrikes’ behavior activated in the magpie’s presence is inborn, this ability was selected only against the magpie, because the magpie has a longer co-evolution with the red-backed shrikes. Shrikes displaying the alternative strategy have a significant evolutionary advantage, while shrikes showing active nest defense have been selected out of the population. 2) The shrikes’ behavior activated in the magpie’s presence is based on the individual experience of particular birds. This presumes the development of a good long-term memory or a specialized, episodic-like memory [77]. The occurrence of an episodic-like memory or a what-where-when memory [78] has never been shown in shrikes. Nevertheless, shrikes are known for their impaling behavior—storing prey on thorns within their territory [79][80], which probably places some demands on their spatial memory skills. 3) The shrikes’ behavior activated in the magpie’s presence is transferred from parents to their offspring or from other shrikes in the population. This explanation also presupposes quite high cognitive abilities in shrikes, which must be involved in the shrike’s antipredator behavior, because the magpie represents a cognitively well-developed adversary. Thus, the two cognitively developed species in mutual combat may represent an interesting model system for the study of the cognitive abilities of birds.

Our results did not show any effect of the order of the dummy presentation. This suggests that there was no reinforcement during the course of four trials. This is seemingly in contrast to our previous results [81], showing that imperfect dummy is attacked more when presented after a perfect one as a results of priming. As the dummies of kestrel in our experiments were equally perfect, we did not show any such effects. Nevertheless, both these results show no effect of habituation, shrikes were always very active, despite the daytime or the time spent attacking the dummies.

Taken together, the existing scientific knowledge finds only small differences in the cognitive abilities and foraging mode of magpies and jays but shrikes respond to their presence with a completely different (and proper) strategy. The behavior of shrikes suggests that the magpie is a more dangerous predator than the jay and this presents new challenges to our understanding of the shrike’s motivation in choosing such different antipredatory strategies in response to such seemingly similar predators.
